# Assessing the quality of life in children with autism spectrum disorder: a cross-sectional study of contributing factors

**DOI:** 10.3389/fpsyt.2024.1507856

**Published:** 2024-12-16

**Authors:** Jaya Shanker Tedla, Faisal Asiri, Ravi Shankar Reddy, Devika Rani Sangadala, Kumar Gular, Venkata Nagaraj Kakaraparthi

**Affiliations:** Program of Physical Therapy, Department of Medical Rehabilitation Sciences, College of Applied Medical Sciences, King Khalid University, Abha, Saudi Arabia

**Keywords:** autism spectrum disorder, quality of life, pediatric, comorbid conditions, socioeconomic status, symptom severity

## Abstract

**Objective:**

The study aimed to assess the quality of life (QoL) in children with autism spectrum disorder (ASD) compared to typically developing peers, identify key influencing factors such as socio-demographic and comorbid conditions, and examine the impact of symptom severity on QoL outcomes.

**Methods:**

In this cross-sectional study conducted in Saudi Arabia, 75 children with ASD were compared to 75 typically developing children matched for age and gender. QoL was evaluated using the Pediatric Quality of Life Inventory (PedsQL), while the severity of autism symptoms was assessed using the Autism Diagnostic Observation Schedule (ADOS). Additional variables, including socio-demographic factors, comorbid conditions, and family environment, were collected through structured interviews and clinical assessments. Statistical analyses, including independent samples t-tests, multiple linear regression, and ANOVA, were employed to compare QoL scores, identify predictors, and assess the impact of symptom severity on QoL outcomes.

**Results:**

The mean overall QoL score for children with ASD was 57.86 (SD = 13.25) compared to 81.67 (SD = 10.89) for typically developing children (t = -10.56, p < 0.001, Cohen’s d = 1.90). Socioeconomic status (β = -0.25, t = -5.00, p < 0.001), comorbid ADHD (β = -0.35, t = -5.83, p < 0.001), and parental mental health issues (β = -0.45, t = -9.00, p < 0.001) were significant predictors of lower QoL. ANOVA results showed that children with severe autism symptoms had the lowest QoL scores (mean = 40.12, SD = 15.67; F = 20.45, p < 0.001, η² = 0.45).

**Conclusion:**

Children with ASD showed significantly lower QoL, particularly in social and school functioning, highlighting the need for targeted interventions addressing core symptoms and environmental and family factors to improve outcomes.

## Introduction

Autism Spectrum Disorder (ASD) is a neurodevelopmental condition characterized by persistent challenges in social communication and interaction, along with restricted and repetitive behaviors or interests (1). Globally, there has been a notable rise in ASD prevalence, with recent estimates indicating approximately 1 in 54 children in the United States diagnosed with ASD (1). This increase has underscored the intricate challenges faced by individuals with ASD and their families (2). The origins of ASD are multifaceted, involving a blend of genetic and environmental factors (2). The diverse spectrum of symptoms and functional challenges associated with ASD significantly influences various aspects of quality of life (QoL), necessitating a comprehensive understanding of these impacts (3).

Comorbidities are highly prevalent in children with ASD and significantly impact their QoL (4). A study by Mutluer et al. (5) reported that up to 50% of children with ASD have co-occurring ADHD, while anxiety disorders affect approximately 40% of this population (6). These conditions exacerbate the challenges faced by children with ASD, leading to more pronounced difficulties in emotional regulation, social interaction, and daily functioning, further impacting their overall quality of life (6). Understanding the prevalence and impact of these comorbidities is essential for developing targeted interventions to improve their well-being, adaptive functioning, and overall quality of life.

The severity of autism symptoms is another critical determinant of QoL in children with ASD. Symptom severity can vary widely among individuals, ranging from mild to severe, significantly impacting daily functioning and overall well-being (7). Children with milder symptoms may have better communication skills and social interactions, allowing for greater participation in typical childhood activities (8). In contrast, children with severe symptoms often experience profound difficulties in communication, social interaction, and behavior, which can lead to increased dependency on caregivers and reduced opportunities for social engagement (9). Studies have shown that higher severity of autism symptoms is associated with lower QoL, particularly in areas of social functioning and adaptive behaviors (9). This relationship underscores the importance of tailored interventions that address the specific needs of children based on the severity of their symptoms (9).

Despite the growing body of research on QoL in children with ASD, significant gaps remain in understanding its multidimensional nature (10). The educational outcomes and peer relationships are often underexplored, despite their critical role in overall well-being (11). Moreover, existing studies rarely examine how symptom severity disproportionately impacts specific QoL domains, such as the pronounced effects on social functioning compared to physical health (11). This study addresses these gaps by employing a comprehensive methodology that evaluates multiple QoL domains, incorporates standardized severity assessments, and considers key factors such as socio-demographics and comorbid conditions (12). By linking symptom severity to domain-specific QoL impacts, this research provides a nuanced understanding of how ASD affects daily life and overall well-being.

Recent research has highlighted critical limitations in traditional QoL measures for children with ASD, including their lack of specificity and cultural adaptability. Simpson et al. (13) review underscores the importance of developing ASD-specific scales to capture the unique challenges faced by this population. Similarly, the study by Chezan et al. (14) demonstrates how advanced statistical methodologies can enhance the validity of QoL assessments in ASD populations (14). Despite these advancements, many studies rely on cross-sectional data, limiting the ability to capture changes in QoL over time. Additionally, the underrepresentation of subgroups such as non-verbal children and the lack of diversity in participant samples have restricted the generalizability of findings, underscoring the need for a more inclusive approach (14). This study addresses these gaps by focusing on a comprehensive assessment of QoL and its determinants in a diverse cohort of children with ASD.

Given the critical importance of QoL for children with ASD, the study objectives were to evaluate the overall QoL in children with ASD and compare it with typically developing children, to identify key factors influencing QoL in children with ASD, including socio-demographic variables, comorbid conditions, and family environment, and to explore the relationship between ASD symptom severity and QoL. Specifically, we hypothesized that (1) children with ASD would have lower overall QoL compared to typically developing peers, (2) socio-demographic factors, comorbid conditions, and family environment would significantly impact QoL in children with ASD, and (3) greater severity of autism symptoms would be associated with lower QoL.

## Materials and methods

### Study setting and design

The study was conducted at Abha Tertiary Children’s Hospital, a leading pediatric care center serving a diverse population in the southern region of Saudi Arabia. The hospital provides specialized neurodevelopmental and general pediatric services, ensuring access to a representative sample of children with ASD and typically developing peers. The demographic diversity of the patient population reflects a range of socioeconomic and cultural backgrounds, which enhances the generalizability of the findings to similar healthcare settings. Ethical guidelines as per the Declaration of Helsinki were strictly followed, including obtaining ethical approval (REC#234-2022) and securing written informed consent from parents, along with assent from the children involved.

### Participants

The study included a total of 150 children, consisting of 75 children diagnosed with ASD and 75 typically developing children who served as a control group. An IQ threshold of 50 was selected for exclusion to minimize confounding effects related to severe intellectual disabilities, which can independently impact QoL and obscure the specific contributions of ASD-related factors (15). This threshold also aligns with previous studies investigating QoL in children with ASD, ensuring comparability of findings. The six-month follow-up period was chosen to ensure clinical stability and consistency in participants’ medical and developmental status, providing a reliable basis for QoL assessment (16). Typically developing children were confirmed through a combination of parent-reported developmental histories, clinical evaluations conducted by pediatricians, and standardized screening tools, such as the Ages and Stages Questionnaire (ASQ). To confirm the diagnosis of ASD, evaluations were conducted by a board-certified child and adolescent psychiatrist with expertise in neurodevelopmental disorders. The diagnosis was made according to the DSM-5 criteria, utilizing standardized diagnostic tools, including the Autism Diagnostic Observation Schedule (ADOS) and the Autism Diagnostic Interview-Revised (ADI-R).

The control group comprised typically developing children recruited from the general pediatric clinic of a specialist hospital. To ensure homogeneity, participants were screened through parent-reported developmental histories, standardized screening tools (Ages and Stages Questionnaire), and clinical evaluations conducted by pediatricians to confirm the absence of any developmental, neurological, or psychiatric conditions. Although matching and strict exclusion criteria were applied, including screening for developmental delays, neurological conditions, and psychiatric disorders using the Ages and Stages Questionnaire, challenges in ensuring complete adherence to these criteria are acknowledged. The reliance on parent-reported histories and clinical evaluations may have contributed to some inconsistencies, which are reflected in the results.

### Variables

The outcome variable in this study was the quality of life (QoL) of children with ASD, assessed using the Pediatric Quality of Life Inventory (PedsQL), which measures QoL across physical health, emotional well-being, social functioning, and school functioning. Independent variables included socio-demographic factors (age, gender, socioeconomic status, and parental education), comorbid conditions (e.g., ADHD, anxiety), family environment (e.g., family structure, parental mental health), and ASD symptom severity. Additional variables assessed included type of schooling (mainstream or special education), daily living skills (independent or assisted), social skills, communication skills, and social inclusion.

The Pediatric Quality of Life Inventory (PedsQL) was used to measure QoL across four domains, using a 23-item Likert scale transformed to a 0–100 scale, with higher scores indicating better QoL. The Autism Diagnostic Observation Schedule, Second Edition (ADOS-2), assessed ASD symptom severity across communication, social interaction, and restricted behaviors. Symptom severity was categorized as mild, moderate, or severe using calibrated severity scores. Socio-demographic and family-related variables were collected through structured parent interviews and medical record reviews, while comorbid conditions were diagnosed based on DSM-5 criteria. Other variables, such as daily living skills, communication, and social skills, were assessed through clinician evaluations and validated questionnaires.

Comorbid conditions, specifically the presence of ADHD and anxiety, were identified through medical records and parent interviews. The presence of these conditions was confirmed using standardized diagnostic criteria from the DSM-5 (17), as recorded in the child’s medical history. Additionally, information regarding the family environment, including family structure (single-parent or two-parent households) and parental mental health status, was collected through structured interviews and reviewed from the medical records.

Parental mental health issues were assessed through structured clinical interviews conducted by trained clinicians, focusing on symptoms of anxiety, depression, and stress. Information was corroborated with medical records where available. Parents were categorized as having mental health issues if they reported clinically significant symptoms or had a formal diagnosis documented in their medical history. The types of therapy received by participants were categorized into three primary modalities: behavioral therapy, speech therapy, and occupational therapy. Many children received combined therapies, a common approach in ASD intervention. For analysis purposes, each child was assigned to the primary therapy type they were receiving most consistently, as reported by parents and verified through clinical records. Participants receiving significant, equally distributed components of two or more therapies were classified under a ‘combined therapy’ category.

### Sample size calculation

The sample size calculation was conducted using G*Power statistical software to ensure sufficient power for detecting meaningful differences between groups. The primary statistical tests considered in the calculation included independent samples t-tests for comparing QoL scores between groups and ANOVA for examining the relationship between ASD symptom severity and QoL. A medium effect size (Cohen’s d = 0.5) was chosen based on previous studies in similar populations, which reported medium to large effect sizes for QoL differences in children with ASD. This effect size was deemed appropriate given the anticipated variability in QoL scores across groups and the study’s focus on identifying clinically significant differences. To achieve a power of 0.80 and a significance level of 0.05, a minimum of 64 participants per group was required, and additional participants were recruited to account for potential dropouts and incomplete data.

### Data analysis

Data were analyzed using IBM SPSS Statistics, Version 24.0. Descriptive statistics summarized the demographic and clinical characteristics of the sample, and the data’s normal distribution validated the use of parametric tests. For Objective 1, independent samples t-tests were used to compare mean QoL scores across all domains between children with ASD and typically developing children. QoL was analyzed as a continuous variable. Multiple linear regression analysis was performed for Objective 2 to identify independent factors associated with QoL, with all predictors, including socio-demographic factors, comorbid conditions, family environment, and ASD symptom severity, entered into the model simultaneously. Beta coefficients (β) indicated the direction and magnitude of relationships, and multicollinearity checks (e.g., variance inflation factor analysis) ensured variables were not excessively correlated. For Objective 3, Pearson correlation coefficients (r) explored the relationship between ASD symptom severity (measured continuously via ADOS) and QoL scores. Regression analysis further evaluated the collective impact of independent variables while controlling for confounding factors. To examine group differences in QoL scores across mild, moderate, and severe ASD symptom categories, ANOVA was used, followed by Tukey’s HSD *post-hoc* tests. All statistical analyses were two-tailed, with a significance level of p < 0.05.

## Results

A total of 150 participants were recruited for the study, comprising 75 children with ASD and 75 typically developing children. The representativeness of the control group may be limited due to the recruitment setting, which was a specialist hospital. Although stringent screening ensured the inclusion of typically developing children, this population may not fully reflect the diversity of typically developing children in the general population. All recruited participants completed the study, resulting in a 100% response rate with no drop-outs. The children in both groups were matched for age and gender, ensuring comparability. The ASD group had a mean age of 10.50 years (SD = 2.50), while the typically developing group had a mean age of 10.30 years (SD = 2.40). The majority of participants in both groups were male, with 66.67% in the ASD group and 64.00% in the typically developing group.

Children with ASD exhibit notable differences in various demographic and clinical characteristics compared to typically developing children (**Table 1**). The age distribution is similar between the two groups, but a higher proportion of children with ASD are male (66.67%) compared to typically developing children (64.00%). Socioeconomic status and parental education levels are comparable between the groups. Notably, children with ASD have higher instances of comorbid conditions, with 66.67% affected, and a significant portion of these children require special education (60.00%) compared to typically developing children, most of whom attend mainstream schools (86.67%). Children with ASD also display lower levels of daily living skills, social skills, and communication skills, with 73.33% needing assistance for daily living and 40.00% being non-verbal. The observed findings in **Table 1** may partially reflect limitations in the rigorous exclusion of children with undiagnosed developmental, neurological, or psychiatric conditions, despite the use of standardized screening tools.

**Table 1:** Demographic and clinical characteristics of children with ASD and typically developing children.

Characteristics	Children with ASD (n=75)	Typically Developing Children (n=75)	p-value
Age (years)	10.50 ± 2.50	10.30 ± 2.40	0.651
Gender (Male/Female)	50/25 (66.67%/33.33%)	48/27 (64.00%/36.00%)	0.732
Socioeconomic Status (Low/Middle/High)	20/40/15 (26.67%/53.33%/20.00%)	18/42/15 (24.00%/56.00%/20.00%)	0.924
Parental Education (High School/College/Graduate)	10/45/20 (13.33%/60.00%/26.67%)	8/47/20 (10.67%/62.67%/26.67%)	0.855
Severity of ASD Symptoms (Mild/Moderate/Severe)	20/40/15 (26.67%/53.33%/20.00%)	N/A	N/A
Comorbid Conditions (Yes/No)	50/25 (66.67%/33.33%)	N/A	N/A
Family Structure (Single-parent/Two-parent)	20/55 (26.67%/73.33%)	15/60 (20.00%/80.00%)	0.612
Parental Mental Health Issues (Yes/No)	30/45 (40.00%/60.00%)	N/A	N/A
Type of Schooling (Mainstream/Special Education)	30/45 (40.00%/60.00%)	65/10 (86.67%/13.33%)	0.001
Therapy Received (Behavioral/Speech/Occupational)	40/25/10 (53.33%/33.33%/13.33%)	N/A	N/A
Daily Living Skills (Independent/Assisted)	20/55 (26.67%/73.33%)	60/15 (80.00%/20.00%)	0.003
Social Skills (High/Medium/Low)	15/40/20 (20.00%/53.33%/26.67%)	40/20/15 (53.33%/26.67%/20.00%)	0.002
Communication Skills (Verbal/Non-verbal)	45/30 (60.00%/40.00%)	70/5 (93.33%/6.67%)	0.011
School Support Services (Yes/No)	35/40 (46.67%/53.33%)	60/15 (80.00%/20.00%)	0.005
Peer Relationships (Good/Average/Poor)	10/30/35 (13.33%/40.00%/46.67%)	45/20/10 (60.00%/26.67%/13.33%)	0.001
Social Inclusion (Yes/No)	50/25 (66.67%/33.33%)	65/10 (86.67%/13.33%)	0.001

ASD, Autism Spectrum Disorder. N/A, Not Available.

Children diagnosed with ASD exhibit markedly diminished QoL in comparison to their typically developing peers across various domains (see **Table 2**). In terms of physical health, the average score for children with ASD was 65.32, significantly lower than the mean score of 85.67 for typically developing children, indicating a mild to moderate effect size of 1.75 and a highly significant t-value of -9.87 (p < 0.001). Similarly, emotional well-being scores were notably lower among children with ASD, averaging 58.76 compared to 78.23 for their typically developing counterparts, with a Cohen’s d of 1.60 and a t-value of -8.76 (p < 0.001). Social functioning showed the most substantial disparity, where children with ASD scored 50.22 on average, in contrast to 82.34 for their typically developing peers, resulting in a Cohen’s d of 2.10 and a t-value of -11.45 (p < 0.001). School functioning followed a similar pattern, with children with ASD scoring 55.13 versus 80.45 for typically developing children, with an effect size of 1.80 and a t-value of -9.34 (p < 0.001). The overall QoL was markedly lower for children diagnosed with ASD, evidenced by a mean score of 57.86 compared to 81.67 for typically developing children. This finding yielded a substantial Cohen’s d of 1.90 and a highly significant t-value of -10.56 (p < 0.001).

**Table 2:** Comparison of overall quality of life between children with ASD and typically developing children

Quality of Life Domains	Children with ASD (Mean ± SD)	Typically Developing Children (Mean ± SD)	t-value	p-value	Cohen’s d
Physical Health	65.32 ± 12.45	85.67 ± 10.12	-9.87	<0.001	1.75
Emotional Well-being	58.76 ± 14.32	78.23 ± 11.45	-8.76	<0.001	1.60
Social Functioning	50.22 ± 15.67	82.34 ± 10.76	-11.45	<0.001	2.10
School Functioning	55.13 ± 13.89	80.45 ± 11.23	-9.34	<0.001	1.80
Overall Quality of Life	57.86 ± 13.25	81.67 ± 10.89	-10.56	<0.001	1.90

ASD, Autism Spectrum Disorder; SD, Standard Deviation; t-value, t-test value; p-value, Probability value; Cohen’s d, Effect size.

**Figure 1:**
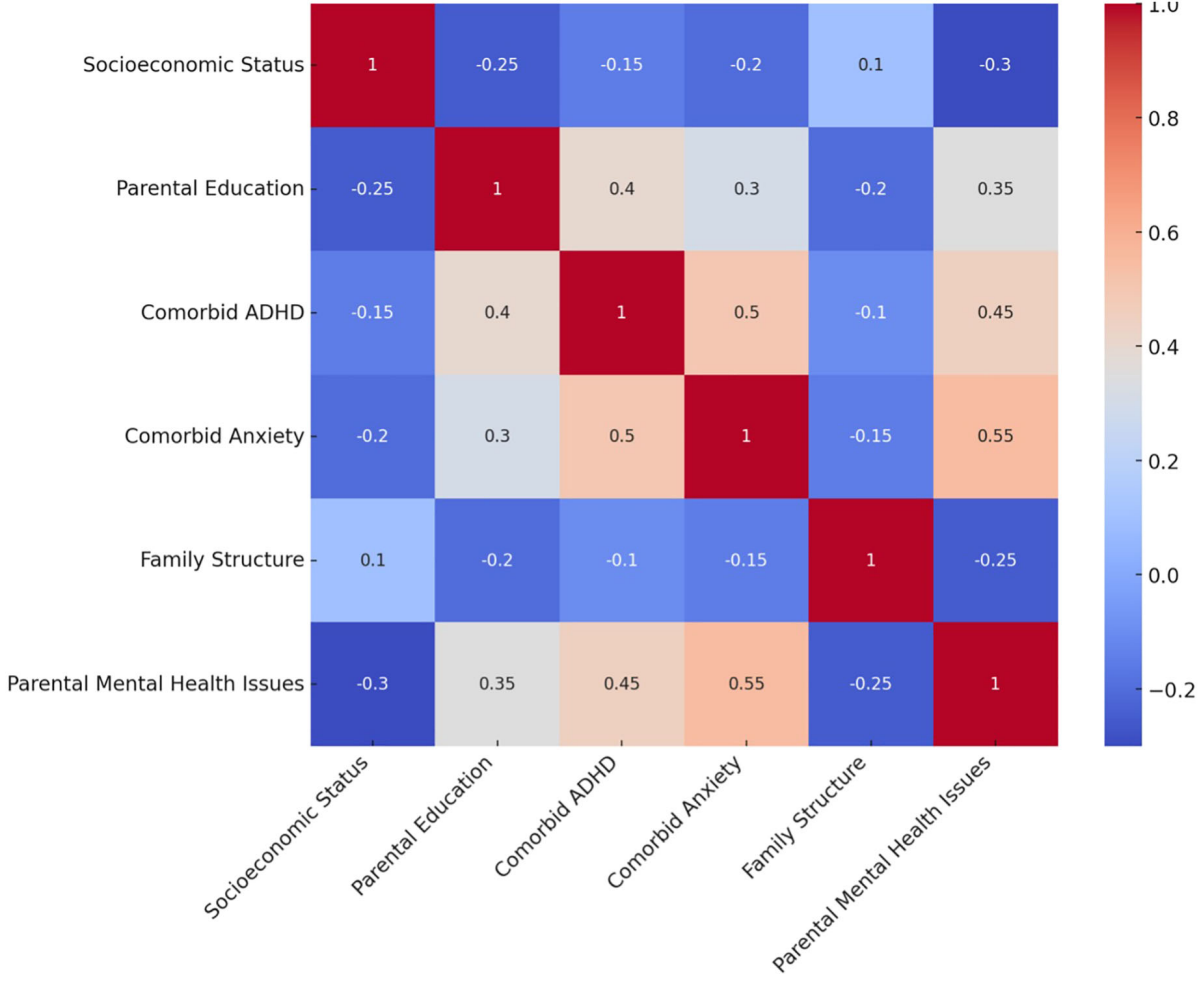
Correlation heatmap of key factors influencing quality of life in children with ASD.

The multiple regression analysis revealed several significant factors influencing the quality of life (QoL) in children with ASD (**Table 3** and **Figure 1**). Lower socioeconomic status (β = -0.25, p < 0.001), comorbid ADHD (β = -0.35, p < 0.001), comorbid anxiety (β = -0.40, p < 0.001), single-parent family structure (β = -0.20, p < 0.001), and parental mental health issues (β = -0.45, p < 0.001) were associated with lower QoL scores. Conversely, higher parental education was positively associated with QoL (β = 0.30, p < 0.001).

**Table 3:** Multiple regression analysis of factors influencing quality of life in children with ASD.

Variable	Regression Coefficient (β)	Standard Error (SE)	t-value	p-value
Socioeconomic Status	-0.25	0.05	-5.00	<0.001
Parental Education	0.30	0.04	7.50	<0.001
Comorbid ADHD	-0.35	0.06	-5.83	<0.001
Comorbid Anxiety	-0.40	0.05	-8.00	<0.001
Family Structure (Single-parent)	-0.20	0.04	-5.00	<0.001
Parental Mental Health Issues	-0.45	0.05	-9.00	<0.001

ASD, Autism Spectrum Disorder; SE, Standard Error; t-value, t-test value; p-value, Probability value; β: Beta Coefficient.​

**Figure 2:**
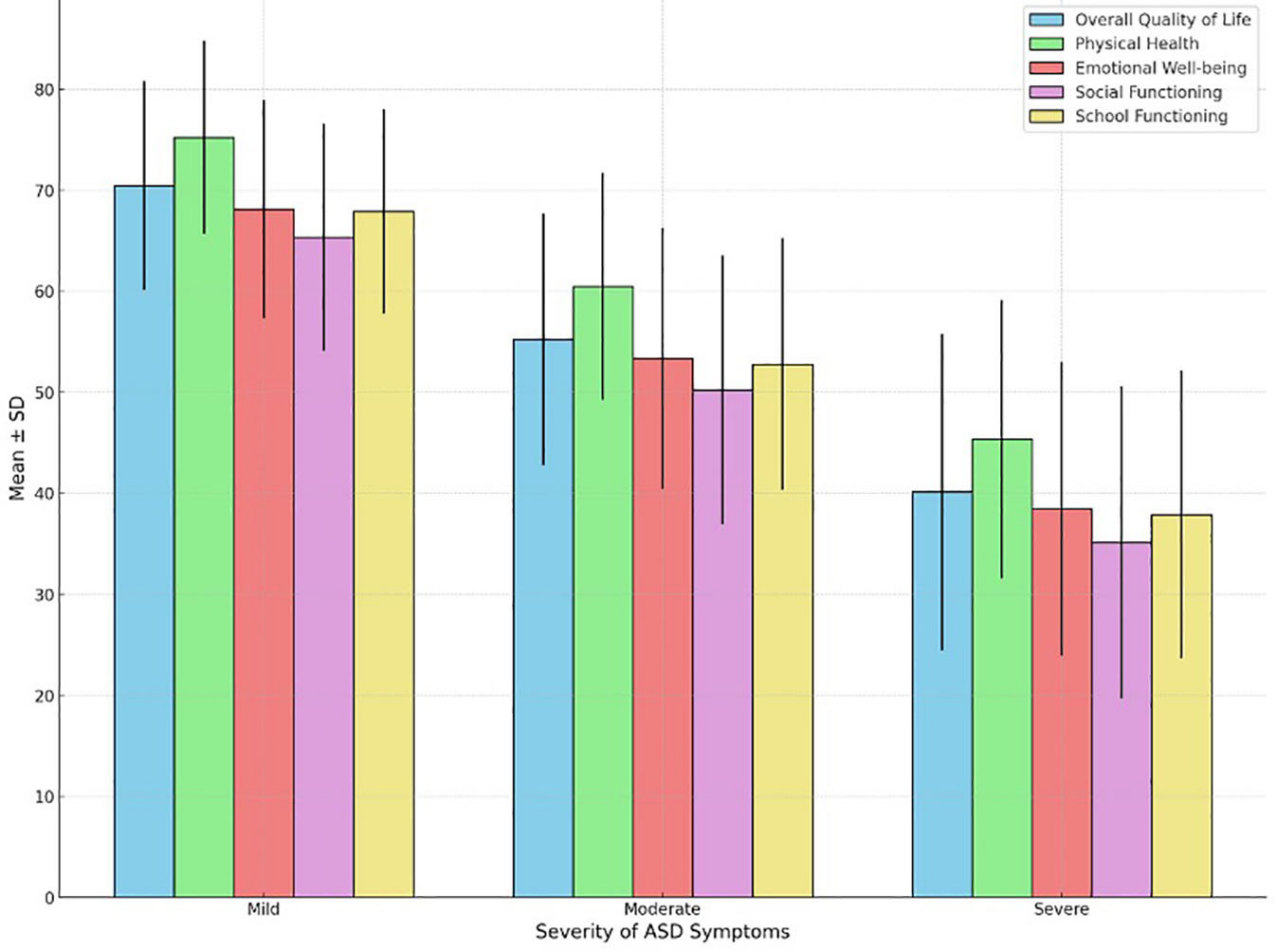
Quality of life in children with ASD by severity of symptoms.

**Table 4** and **Figure 2** provides detailed insights into the relationship between the severity of autism symptoms and the QoL in children diagnosed with ASD. Children with mild ASD symptoms exhibit notably higher overall QoL (mean = 70.45) compared to those with moderate (mean = 55.23) and severe symptoms (mean = 40.12), with all comparisons demonstrating strong statistical significance (p < 0.001). Scores for physical health, emotional well-being, social functioning, and school functioning decline progressively with increasing symptom severity. Effect sizes (η²) ranging from 0.35 to 0.45 underscore the substantial impact of symptom severity on these QoL domains.

**Table 4:** Relationship between severity of autism symptoms and quality of life in children with ASD.

Severity of ASD Symptoms	Number of Children (n)	Overall Quality of Life (Mean ± SD)	Physical Health (Mean ± SD)	Emotional Well-being (Mean ± SD)	Social Functioning (Mean ± SD)	School Functioning (Mean ± SD)	F-value	p-value	Effect Size (η²)
Mild	25	70.45 ± 10.34	75.23 ± 9.56	68.12 ± 10.78	65.34 ± 11.23	67.89 ± 10.12	15.34	<0.001	0.35
Moderate	30	55.23 ± 12.45	60.45 ± 11.23	53.34 ± 12.90	50.23 ± 13.34	52.78 ± 12.45	18.23	<0.001	0.40
Severe	20	40.12 ± 15.67	45.34 ± 13.78	38.45 ± 14.56	35.12 ± 15.45	37.89 ± 14.23	20.45	<0.001	0.45

ASD, Autism Spectrum Disorder; SD, Standard Deviation; F-value, F-test value; p-value, Probability value; η², Eta Squared (Effect Size).

## Discussion

In this study, we aimed to assess the QoL in children with ASD and compare it with typically developing children, identify key factors influencing their QoL, and explore the relationship between the severity of autism symptoms and QoL. The differences in mean QoL scores between children with ASD and typically developing peers were statistically significant across all domains, with p-values <0.001. However, the effect sizes, as measured by Cohen’s d, ranged from 0.60 to 0.79, indicating moderate effects rather than large effects, based on standard guidelines. These moderate effect sizes suggest that while the differences are meaningful, other factors may contribute to QoL disparities and warrant further exploration. Key factors influencing the QoL in children with ASD include socioeconomic status, parental education, comorbid ADHD and anxiety, family structure, and parental mental health issues, all of which were significantly associated with variations in QoL. Furthermore, the severity of autism symptoms was inversely related to QoL, with children experiencing mild symptoms reporting higher QoL than those with moderate or severe symptoms, highlighting the substantial impact of symptom severity on physical health, emotional well-being, social functioning, and school functioning. These findings emphasize the need for targeted interventions to address these factors and improve the QoL for children with ASD.

The findings indicate that children with ASD experience a significantly lower QoL across various domains, including physical health, emotional well-being, social functioning, and school functioning, compared to typically developing children (18). Several factors may contribute to these disparities. Children with ASD often face challenges in physical health due to co-occurring conditions, dietary restrictions, and lower levels of physical activity (19). Emotional well-being is impacted by the higher prevalence of anxiety, depression, and difficulty in processing emotions (20). Social functioning deficits arise from impaired social communication skills and difficulties in forming peer relationships (21). School functioning is adversely affected by the need for specialized educational support and the challenges in coping with the academic and social demands of a mainstream school environment (22). These cumulative challenges significantly lower the overall QoL for children with ASD. These results are consistent with previous studies that have documented similar findings. For instance, Kuhlthau et al. (2010) reported that children with ASD have poorer health-related QoL compared to their peers, primarily due to social and emotional difficulties (23). Additionally, research by Eapen et al. (24) highlighted the impact of comorbid conditions such as anxiety and ADHD on the overall QoL in children with ASD (24). The findings of our study align with these previous observations, underscoring the need for comprehensive interventions that address not only the core symptoms of ASD but also the associated physical, emotional, and social challenges to improve the QoL for these children (25).

The study identifies several key factors that significantly influence the QoL in children with ASD. Socioeconomic status is a notable factor, with lower socioeconomic status correlating with reduced QoL, likely due to limited access to resources, healthcare, and educational opportunities (26). Higher parental education levels are associated with better QoL, possibly because educated parents are better equipped to navigate the healthcare and educational systems to secure necessary services for their children (27). Comorbid conditions, particularly ADHD and anxiety, substantially reduce the QoL due to their additional psychological and behavioral burdens (28). The impact of family structure is evident, with children from single-parent families experiencing a lower QoL, likely due to reduced social and financial support (29). Furthermore, parental mental health issues significantly decrease the QoL in children with ASD, as these issues can affect the overall family environment and the level of support provided to the child (30). These findings are consistent with previous research, further validating the results of this study. For instance, Mathew et al. (31) found that children with ASD from lower socioeconomic backgrounds had poorer health outcomes and reduced QoL, highlighting the influence of socioeconomic status (31). Similarly, Koukouriki et al. (32) reported that higher parental education levels are linked to better health-related QoL in children with ASD, supporting the current study’s findings (32). The detrimental effects of comorbid conditions such as ADHD and anxiety on QoL have been well-documented by D’Agati et al. (33), who emphasized the additional challenges these conditions pose (33). The impact of family structure is also supported by research from Blackstock et al. (34), who observed that single-parent families face more significant difficulties, affecting the child’s well-being (34). Lastly, the influence of parental mental health issues on child outcomes has been substantiated by studies like those of Martin et al. (35), which underscore the importance of addressing parental mental health to improve the QoL in children with ASD (35).

The study reveals a clear relationship between the severity of autism symptoms and the QoL in children with ASD (36). Children with mild ASD symptoms report a significantly higher overall QoL (mean = 70.45) compared to those with moderate (mean = 55.23) and severe symptoms (mean = 40.12), with all comparisons showing strong statistical significance (p < 0.001). As symptom severity increases, scores in physical health, emotional well-being, social functioning, and school functioning all decline markedly (37). This trend can be attributed to the greater challenges faced by children with more severe symptoms, including more pronounced difficulties in communication, social interaction, and behavior, which in turn affect their ability to engage in daily activities, form relationships, and succeed in academic settings (38). The effect sizes (η²) range from 0.35 to 0.45, indicating a substantial impact of symptom severity on these domains of QoL. These findings are corroborated by previous research. For instance, Yerys et al. (36) found that higher severity of autism symptoms is associated with lower QoL, particularly in areas of social functioning and adaptive behaviors (36). Similarly, studies by Stole et al. (39) and Young et al. (40) support the notion that children with more severe autism symptoms experience greater difficulties that negatively impact their overall well-being and daily functioning (39, 40). These studies highlight the cumulative burden of severe autism symptoms on various aspects of life, emphasizing the need for targeted interventions that address the specific needs of children based on their symptom severity (41). This alignment with previous research underscores the reliability and validity of the current study’s findings, reinforcing the importance of a nuanced approach to support and intervention for children with ASD.

The findings of this study align with prior research, such as those by Eapen et al. (42) and Mavroeidi et al. (43), which reported lower QoL in children with ASD compared to typically developing peers. However, the unique socio-cultural context of the study population in Saudi Arabia may influence generalizability. Cultural factors, including familial structures and societal stigma, can shape the experiences of children with ASD differently than in Western settings. The extended family support networks in Saudi Arabia may enhance emotional well-being, while limited access to specialized services exacerbates challenges in school and social functioning. Comparisons with Western studies, where inclusive policies and broader resources often lead to better school functioning outcomes, highlight the need for region-specific strategies. Future research should examine QoL across diverse socio-cultural settings to better understand the influence of contextual factors.

This study highlights the urgent need for targeted interventions to address the significantly lower QoL observed in children with ASD compared to their typically developing peers. Key findings emphasize the importance of addressing socioeconomic disparities through equitable access to resources, such as subsidized special education, inclusive teacher training, and individualized education plans. Community-based programs, including parent-led support groups and peer-mediated social skills interventions, can further bridge gaps in service access. To mitigate the impact of parental mental health issues, family-centered approaches such as mental health counseling, stress management workshops, respite care, and strengthened community support are essential. The influence of socioeconomic status, parental education, comorbid conditions, and family environment underscores critical areas for clinical focus, while the correlation between symptom severity and QoL highlights the need for personalized treatment plans.

This study provides valuable insights into the QoL of children with ASD, but several limitations should be noted. The reliance on parent-reported QoL measures may introduce subjective bias, as parents’ perceptions may not fully capture the child’s lived experiences. Although standardized tools like the PedsQL were used to mitigate this, future studies should incorporate direct child assessments for a more comprehensive perspective. The sample was exclusively drawn from Abha Tertiary Children’s Hospital, limiting the generalizability of findings to other regions or healthcare settings and potentially missing broader cultural and socioeconomic diversity. The study did not evaluate specific ASD symptoms, such as restrictive behaviors or sensory issues, which are critical determinants of QoL, limiting the depth of insights. Additionally, the recruitment of typically developing children from the outpatient clinic of a specialist hospital may not reflect the general population, and challenges in rigorously excluding undiagnosed developmental or psychiatric conditions remain. Future research should include community-based recruitment, more comprehensive assessments, and broader population diversity to enhance representativeness and understanding.

## Conclusion

In conclusion, this study provides compelling evidence that children with ASD experience a significantly lower QoL compared to their typically developing peers, across all measured domains including physical health, emotional well-being, social functioning, and school functioning. Key factors such as socioeconomic status, parental education, comorbid ADHD and anxiety, family structure, and parental mental health issues substantially influence these outcomes. Additionally, the severity of autism symptoms is inversely related to QoL, with greater symptom severity correlating with more pronounced impairments. These findings highlight the critical need for targeted, multifaceted interventions that address both the core symptoms of ASD and the associated socio-demographic and familial factors, to enhance the overall well-being and life satisfaction of children with ASD.

## Data Availability

The data supporting the findings of this study are available in the “ZENODO” repository at DOI: 10.5281/zenodo.13381005.
